# Effect of hydrogen sulfide on inflammatory cytokines in acute myocardial ischemia injury in rats

**DOI:** 10.3892/etm.2015.2218

**Published:** 2015-01-26

**Authors:** FANG LIU, GUANG-JIE LIU, NA LIU, GANG ZHANG, JIAN-XIN ZHANG, LAN-FANG LI

**Affiliations:** 1 Department of Thoracic Surgery, The Fourth Hospital of Hebei Medical University, Shijiazhuang, P.R. China; 2 Department of Gland Surgery, Dingzhou City People’s Hospital, Dingzhou, P.R. China; 3 Department of Gastroenterology, Dingzhou City People’s Hospital, Dingzhou, P.R. China; 4 Department of Pharmacology, Hebei Academy of Medical Sciences, Shijiazhuang, Hebei, P.R. China

**Keywords:** hydrogen sulfide, acute myocardial ischemia, rat, inflammatory factor

## Abstract

Hydrogen sulfide (H_2_S) is believed to be involved in numerous physiological and pathophysiological processes, and now it is recognized as the third endogenous signaling gasotransmitter, following nitric oxide and carbon monoxide; however, the effects of H_2_S on inflammatory factors in acute myocardial ischemia injury in rats have not been clarified. In the present study, sodium hydrosulfide (NaHS) was used as the H_2_S donor. Thirty-six male Sprague Dawley rats were randomly divided into five groups: Sham, ischemia, ischemia + low-dose (0.78 mg/kg) NaHS, ischemia + medium-dose (1.56 mg/kg) NaHS, ischemia + high-dose (3.12 mg/kg) NaHS and ischemia + propargylglycine (PPG) (30 mg/kg). The rats in each group were sacrificed 6 h after the surgery for sample collection. Compared with the ischemia group, the cardiac damage in the rats in the ischemia + NaHS groups was significantly reduced, particularly in the high-dose group; in the ischemia + PPG group, the myocardial injury was aggravated compared with that in the ischemia group. Compared with the ischemia group, the levels of interleukin (IL)-1β, IL-6 and tumor necrosis factor-α (TNF-α) in the serum of rats in the ischemia + medium- and high-dose NaHS groups were significantly reduced, and the expression of intercellular adhesion molecule-1 (ICAM-1) mRNA and nuclear factor κ-light-chain-enhancer of activated B cells (NF-κB) protein in the myocardial tissues of rats was significantly reduced. In the ischemia + PPG group, the TNF-α, IL-1β and IL-6 levels in the serum were significantly increased, the expression of ICAM-1 mRNA was increased, although without a significant difference, and the expression of NF-κB was increased. The findings of the present study provide novel evidence for the dual effects of H_2_S on acute myocardial ischemia injury via the modulation of inflammatory factors.

## Introduction

Myocardial ischemia refers to the absolute or relative lack of coronary blood supply, or transient or chronic myocardial ischemia caused by interruption to the coronary blood supply and hypoxia. This ischemia leads to metabolic disorder of myocardial cells and the accumulation of metabolites, thus causing myocardial injury, or even myocardial necrosis, and thereby affecting cardiac function. Myocardial ischemia clinically manifests as syndromes such as angina pectoris and myocardial infarction. Long-term myocardial ischemia can result in cardiac fibrosis and enlargement of the heart, causing arrhythmia or heart failure, and even resulting in mortality; therefore, it is a serious threat to human health (1).

For hundreds of years, it has been believed that hydrogen sulfide (H_2_S) is a colorless toxic gas with a smell of rotten eggs, which, when over-inhaled, can suppress the central nervous and respiratory systems. Studies on H_2_S have been confined to its toxic effect (2,3); however, since the mid-1990s, when it began to be recognized that H_2_S could promote long-term potentiation in the hippocampus (4,5), there has been increasing evidence that the gas has an important physiological role in the body (6), particularly in the cardiovascular and central nervous systems (7,8). H_2_S is the third novel gaseous signaling molecule, following nitric oxide and carbon monoxide (9,10). In mammals, the endogenous H_2_S is mainly generated by the metabolism of sulfur-containing amino acids, such as L-cysteine; cystathionine-β-synthase (CBS) and cystathionine-γ-lyase (CSE) are the key enzymes in H_2_S generation (7). It has been found that H_2_S not only exerts cardiovascular effects in the cardiovascular system, such as relaxation of vascular smooth muscle, lowering blood pressure, inhibition of vascular smooth muscle cell proliferation and regulation of cardiac contractility, but also is involved in pathophysiological processes, such as hypertension, pulmonary hypertension, acute myocardial infarction and ischemia/reperfusion injury. The incidence and development of myocardial ischemia are complex (11–17). In a previous model, H_2_S was found to exert anti-inflammatory effects (18). It has been reported that, in a myocardial ischemia/reperfusion model, the protective effect of sodium hydrosulfide (NaHS) on myocardial tissues is associated with its anti-inflammatory effects (19; however, it remains unclear whether the protective effect of H_2_S in rats with acute myocardial ischemia is associated with its regulation of inflammatory cytokines. In the present study, therefore, an animal model of acute myocardial ischemia was established in rats by ligation of the coronary artery, in order to observe the effects of the H_2_S donor NaHS and the CSE inhibitor propargylglycine (PPG) on inflammatory cytokines, such as tumor necrosis factor-α (TNF-α), interleukin-1β (IL-1β), IL-6 and nuclear factor κ-light-chain-enhancer of activated B cells (NF-κB), and intercellular adhesion molecule-1 (ICAM-1) in the presence of myocardial ischemia. Furthermore, the effect of H_2_S in rats with acute myocardial ischemia was explored, as well as the possible underlying mechanism.

## Materials and methods

### Drugs and reagents

NaHS and PPG were purchased from Sigma (St. Louis, MO, USA); primers for ICAM-1 and β-actin were obtained from Shanghai Generay Biological Engineering Co., Ltd. (Shanghai, China); the SV Total RNA Isolation system, as well as TaqDNA polymerase, agarose and ethidium bromide, were purchased from Promega Corp. (Madison, WI, USA); the RevertAid First Strand cDNA Synthesis kit used for reverse transcription (RT) was purchased from Thermo Scientific (Waltham, MA, USA); DNA marker was obtained from Beijing SBS Genetech Co., Ltd. (Beijing, China). The polymerase chain reaction (PCR) primers, which were synthesized by Shanghai Generay Biological Engineering Co., Ltd., were as follows: ICAM-1 sense, 5′-AAGGTGTGATATCCGGTAGA-3′ and antisense, 5′-CCTTCTAAGTGGTTGGAACA-3′, β-actin sense, 5′-CGTTGACATCCGTAAAGAC-3′ and antisense, 5′-CTGGAAGGTGGACAGTGAG-3′. A nuclear protein/plasma protein extraction kit was purchased from Beijing Chong League International Biological Gene Technology Co., Ltd. (Beijing, China); rabbit anti-rat NF-κB p65 polyclonal antibody was obtained from Santa Cruz Biotechnology, Inc. (sc-109; 1,100; Santa Cruz, CA, USA); rat β-actin polyclonal antibody (sc-130657) was also obtained from Santa Cruz Biotechnology, Inc. and rat serum TNF-α, IL-1β and IL-6 ELISA detection kits were purchased from R&D Systems, Inc. (Minneapolis, MN, USA).

### Experimental animals

Healthy male Sprague Dawley (SD) rats weighing 270±20 g were provided by the Experimental Animal Center of Hebei Province (Shijiazhuang, China). The present study was approved by the Ethics Committee of The Fourth Hospital of Hebei Medical University (Shijiazhuang, China).

### Experimental models and animal grouping

Thirty-six male SD rats were randomly divided into sham surgery, ischemia, ischemia + low-, middle- and high-dose NaHS and ischemia + PPG groups (n=6). The acute myocardial ischemia model was established by ligating the left anterior descending coronary artery (LAD) of the rats. In the sham surgery group, the LADs were not ligated but only threaded. Saline was intraperitoneally administered to the rats in the ischemia group. In the ischemia + low-, middle- and high-dose NaHS groups and the ischemia + PPG group, NaHS (0.78, 1.56 or 3.12 mg/kg) or PPG (30 mg/kg), respectively, was intraperitoneally injected 3 h after the induction of ischemia. The rats were sacrificed 6 h after the surgery.

### Detection indicators and methods

#### Observation of morphological changes in myocardial tissue by transmission electron microscopy (TEM)

At the end of the ischemia, apical tissues were taken rapidly, rinsed with normal saline to remove the blood, cut into small slices measuring 1×1×1 mm and placed on ice. The samples were then fixed in 4% glutaraldehyde, rinsed twice with 0.1 mol/l cacodylate buffer (Yongda Chemical Reagent Co., Ltd., Tianjin China), fixed with 1% osmium tetroxide and then washed with buffer. The samples were subsequently progressively dehydrated in acetone, impregnated in epoxy, embedded, cut into ultrathin slices and then stained in uranyl acetate-lead citrate. Changes in the myocardial ultrastructure were observed through TEM.

#### Determination of TNF-α, IL-6 and IL-1β levels in the serum

At the end of the ischemia, blood was taken from rats in each group via the right carotid artery, and serum was separated through centrifugation at 1,006 × g for 15 min at 4°C. Double-antibody sandwich ELISA was employed for the detection of TNF-α, IL-6 and IL-1β levels in the serum, in accordance with the manufacturer’s instructions (R&D Systems, Inc.). Optical density values were determined by ELISA and the standard curve was drawn to calculate TNF-α, IL-6 and IL-1β concentrations in the sample.

#### Detection of ICAM-1 mRNA expression in myocardial tissue by semi-quantitative RT-PCR

The RNA extraction kit was used to extract total RNA from the myocardial tissues, and RNA then served as a template to obtain cDNA by RT with the RT-PCR kit (Promega Corp.). β-actin served as a reference gene. The 50-μl PCR reaction system comprised 25 μl Go Taq^®^ Green Master Mix, 1 μl upstream primer, 1 μl downstream primer, 4 μl DNA template and 19 μl nuclease-free water. The reaction conditions were as follows: Initial denaturation at 94°C for 4 min; 35 cycles of 94°C for 45 sec, 60°C for 60 sec and 72°C for 90 sec; 72°C for a further 7 min. The PCR product was analyzed using electrophoresis in a 1% agarose gel and then placed in a gel image analysis system (T-05×20-2A; Vilber Lourmat Co., Marne-la-Vallee, France) for an absorbance scan. β-actin served as a reference for calibration, and the ratio of the absorbance of the target genes to that of β-actin suggested the relative expression levels of the target genes.

#### Detection of NF-κB expression in myocardial tissues by western blotting

The cell lysate was added into myocardial tissues that had been cut and nuclear proteins were extracted in accordance with the kit manufacturer’s instructions (Beijing Chong League International Biological Gene Technology Co., Ltd.). The bicinchoninic acid assay was used to measure protein concentration. Nuclear protein samples were taken, analyzed by the method of gel electrophoresis in 10% sodium dodecyl sulfate-polyacrylamide, and then electrically transferred to a polyvinylidene difluoride membrane. The samples were subsequently mixed with anti-NF-κB p65 polyclonal antibody (1:100 dilution)/β-actin polyclonal antibody (1:500) and kept at 4°C overnight. Following incubation, chemiluminescence, developing and fixing were performed. AlphEaseFC™ software (Alpha Innotech, San Leandro, CA, USA) was employed to analyze the results, and the ratio of the gray value of each target band to that of β-actin protein was provided to analyze the protein of interest.

#### Statistical analysis

Experimental data are presented as the mean ± standard error of the mean. SPSS 13.0 software (SPSS, Inc., Chicago, IL, USA) processing was used for statistical analysis. Comparisons were conducted using one-way analysis of variance, and P<0.05 was considered to indicate a statistically significant difference.

## Results

### Ultrastructural changes in the myocardial tissue

**Figure 1:**
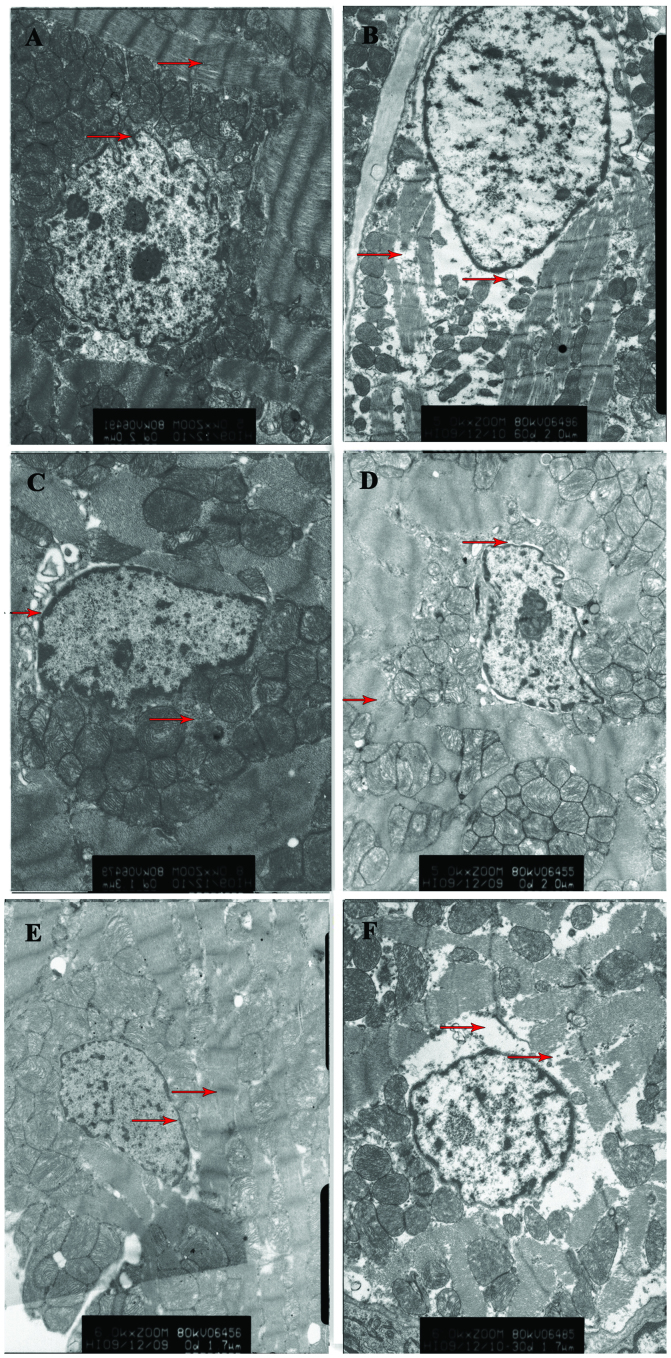
Effect of hydrogen sulfide on the pathological changes in the ultrastructure of the myocardium, as determined by transmission electron microscopy. (A) Sham surgery group rats underwent surgical procedures but without the ischemic insult, followed by treatment with saline. (B) Ischemia group rats underwent surgery and were then treated with saline; the rats showed myocardial fiber disarray and severe edema in the karyoplasm and perinuclear space. (C-E) In the ischemic rats treated with (C) 0.78, (D) 1.56 and (E) 3.12 mg/kg NaHS, the myocardial fiber disarray and nuclear edema were reduced, particularly in the high-dose NaHS group (E). (F) In the ischemic rats treated with 30 mg/kg propargylglycine, the myocardial injury was aggravated. Arrows indicate myocardial fibers and nuclear peripheral tissues; scale, 5,000 nm. NaHS, sodium hydrosulfide.

In the rats from the sham surgery group, neatly arranged myocardial fibers, integrated mitochondrial cristae and membranes and a slight expansion of the perinuclear space were observed. In rats from the ischemia group, it was noted that there was myocardial fiber disarray, severe edema in the karyoplasm and perinuclear space and partial disappearance of the nuclear membrane; there was also severe swelling, deformation and dissolution and disappearance of the mitochondrial cristae and membrane. Compared with that in the ischemia group, the cardiac damage in the ischemia + low-, medium- and high-dose NaHS groups was significantly reduced, particularly in the high-dose group; slightly disordered muscle fiber arrangement and mild edema in the mitochondrial matrix were also observed. In the ischemia + PPG group, the degree of myocardial injury was aggravated compared with that in the ischemia group (Fig. 1).

### Changes in TNF-α, IL-1β and IL-6 levels in the serum

**Figure 2:**
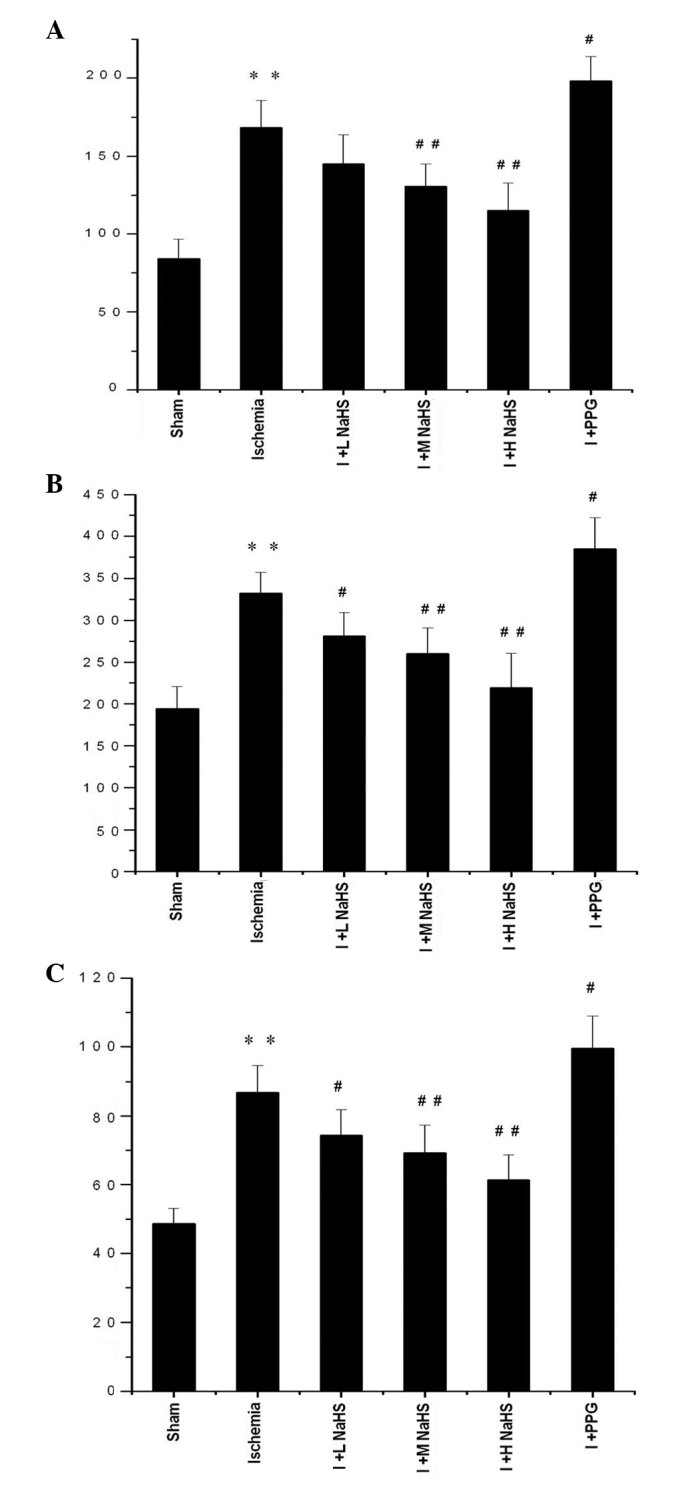
Effect of hydrogen sulfide on the change in the serum levels of (A) tumor necrosis factor-α, (B) IL-6 and (C) IL-1β in rats. Data are presented as the mean ± standard error of the mean (n=6) with the units of ng/l. ^**^P<0.01 vs. the sham group; ^#^P<0.05 and ^##^P<0.01 vs. the ischemia group. Sham, rats underwent the surgical procedures but without the ischemic insult, followed by treatment with saline; Ischemia, rats underwent the surgical procedures and were then treated with saline; I + L NaHS, ischemic rats treated with 0.78 mg/kg NaHS; I + M NaHS, ischemic rats treated with 1.56 mg/kg NaHS; I + H-NaHS, ischemic rats treated with 3.12 mg/kg NaHS; I + PPG, ischemia rats treated with 30 mg/kg PPG; NaHS, sodium hydrosulfide; PPG, propargylglycine; IL, interleukin.

Compared with the sham surgery group, the TNF-α, IL-1β and IL-6 serum levels in the rats were significantly elevated in the ischemia group (P<0.01). Compared with the ischemia group, the IL-1β and IL-6 serum levels were significantly reduced in the ischemia + low-, medium- and high-dose NaHS groups; in the ischemia + medium- and high-dose NaHS groups the TNF-α level in the serum was significantly reduced. In the ischemia + PPG group, the serum levels of TNF-α, IL-1β and IL-6 were significantly increased compared with those in the ischemia group (P<0.05 or P<0.01) (Fig. 2 and Table I).

### Changes in ICAM-1 mRNA expression in the myocardial tissue

**Figure 3:**
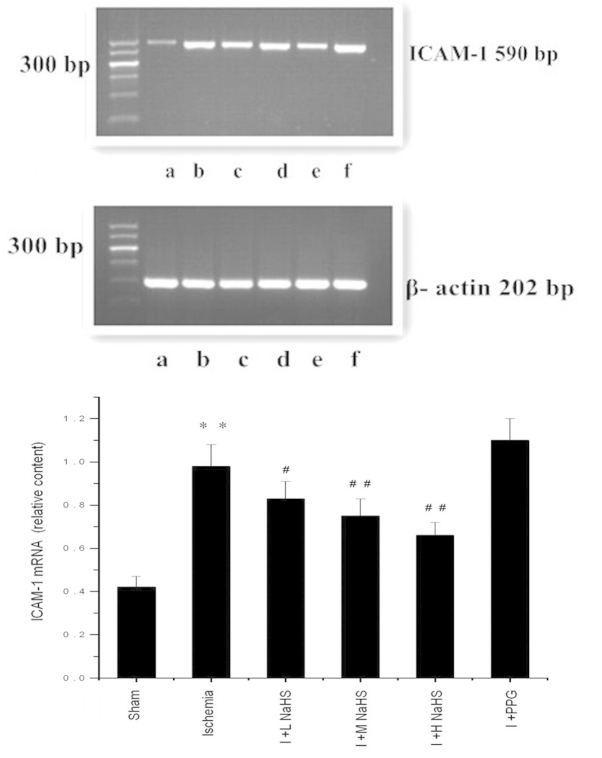
Effect of hydrogen sulfide on the change in the expression of ICAM-1 mRNA in myocardial tissue in rats, as assessed using reverse transcription-polymerase chain reaction analysis: (a) Sham, (b) ischemia, (c) I + L NaHS, (d) I + M NaHS, (e) I + H NaHS, (F) I + PPG. Data are presented as the mean ± standard error of the mean (n=5). ^**^P<0.01 vs. the sham group; ^#^P<0.05 and ^##^P<0.01 vs. the ischemia group. Sham, rats underwent the surgical procedures but without the ischemic insult, followed by treatment with saline; Ischemia, rats underwent the surgical procedures and were then treated with saline; I + L NaHS, ischemic rats treated with 0.78 mg/kg NaHS; I + M NaHS, ischemic rats treated with 1.56 mg/kg NaHS; I + H-NaHS, ischemic rats treated with 3.12 mg/kg NaHS; I + PPG, ischemia rats treated with 30 mg/kg PPG; NaHS, sodium hydrosulfide; PPG, propargylglycine; ICAM-1, intercellular adhesion molecule-1.

In the ischemia group the ICAM-1 mRNA expression in the myocardial tissues of the rats was significantly increased compared with that in the sham surgery group (P<0.01). Compared with the ischemia group, the ICAM-1 mRNA expression in the myocardial tissues of the rats was reduced in the ischemia + low-, medium- and high-dose NaHS groups (P<0.05 or P<0.01); ICAM-1 mRNA expression was increased markedly, but not significantly, in the ischemia + PPG group (P>0.05) (Fig. 3 and Table II).

### Changes in NF-κB expression in the myocardial tissue

**Figure 4:**
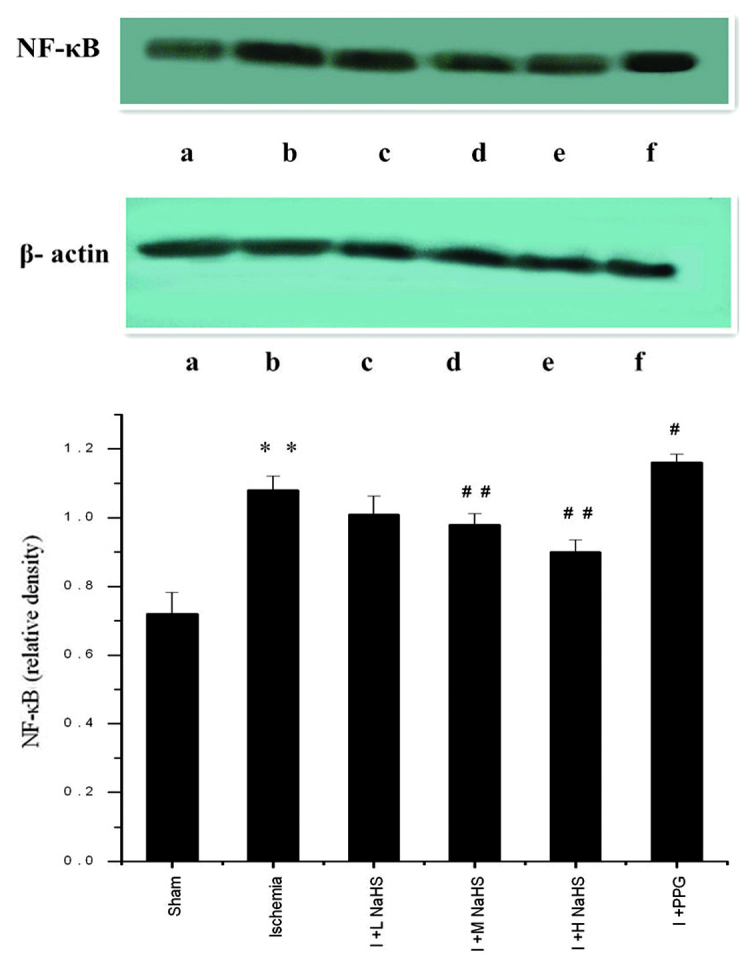
Effect of hydrogen sulfide on the change in the expression of NF-κB in myocardial tissue in rats, as assessed by western blotting: (a) Sham, (b) ischemia, (c) I + L NaHS, (d) I + M NaHS, (e) I + H NaHS, (F) I + PPG. Data are presented as the mean ± standard error of the mean (n=5). ^**^P<0.01 vs. the sham group; ^#^P<0.05 and ^##^P<0.01 vs. the ischemia group. Sham, rats underwent the surgical procedures but without the ischemic insult, followed by treatment with saline; Ischemia, rats underwent the surgical procedures and were then treated with saline; I + L NaHS, ischemic rats treated with 0.78 mg/kg NaHS; I + M NaHS, ischemic rats treated with 1.56 mg/kg NaHS; I + H-NaHS, ischemic rats treated with 3.12 mg/kg NaHS; I + PPG, ischemia rats treated with 30 mg/kg PPG; NaHS, sodium hydrosulfide; PPG, propargylglycine; NF-κB, nuclear factor κ-light-chain-enhancer of activated B cells.

Western blotting results showed a trace amount of NF-κB expression in the myocardial tissues of rats in the sham surgery group; in the ischemia group the NF-κB expression in the myocardial tissues of the rats was significantly increased compared with the sham surgery group (P<0.01). Compared with the ischemia group, NF-κB expression in the myocardial tissues of the rats was significantly reduced in the ischemia + medium- and high-dose NaHS groups; in the ischemia + PPG group, NF-κB expression in the myocardial tissues of the rats was increased (P<0.05 or P<0.01) (Fig. 4 and Table II).

## Discussion

Previously it has been found that numerous mammalian cells and tissues can produce H_2_S, a novel type of gas neurotransmitter in the body with a wide range of biological effects (4,20,21). H_2_S is predominantly generated by L-cysteine under the action of CBS and CSE (13). Numerous mammalian cells, tissues, organs and systems can produce H_2_S, which is mainly synthesized by tissue-specific metabolic enzymes utilizing endogenous methionine, homocysteine and L-cysteine; a small amount of H_2_S is generated by non-enzymatic synthesis (7,23). Endogenous H_2_S is generated in mammals in three main ways, two of which are pyridoxal 5′-phosphate-dependent enzyme regulating pathways. In these pathways, two key enzymes, CBS and CSE, generate H_2_S, pyruvate and ammonium via a transfer action with L-cysteine and homocysteine serving as a substrate (7). The third method of generating H_2_S is through the zinc-dependent 3-mercaptopyruvate sulfurtransferase (3MST) catalytic pathway: Aspartate aminotransferase metabolizes L-cysteine to produce 3-mercaptopyruvate, which is then desulfurized by 3MST to generate H_2_S (24). 3MST is present in the cytoplasm and mitochondria, while CBS and CSE exist only in the cytoplasm. In mammals, the distribution of CBS and CSE is tissue-specific, with CSE found mainly in the cardiac and vascular smooth muscle (14,25) and CBS mainly in the nervous system (26); however CBS and CSE may be expressed simultaneously in the small intestine, liver and kidney (25,27).

One-third of the total H_2_S is present in gaseous form in the body while two-thirds are present in the form of NaHS, which combines with H^+^ in the body to generate H_2_S. A dynamic equilibrium exists between NaHS and HS^−^, so as to ensure the stable presence of H_2_S and the maintenance of the pH of the environment (27). Under physiological conditions, levels of H_2_S in SD rat plasma are ~46 μmol/l (28).

According to the literature (29,30) and the results of the preliminary experiment, the rats in the present study were intraperitoneally injected with 0.78, 1.56 or 3.12 mg/kg NaHS or 30 mg/kg PPG (CSE inhibitor) 3 h after acute myocardial ischemia. NaHS, an H_2_S donor, dissociates into Na^+^ and HS^−^ in aqueous solution, and HS^−^ binds with H^+^ to generate H_2_S (31). Preliminary experiments showed that NaHS and PPG in the above-mentioned doses exerted superior treatment and aggravation effects on acute myocardial ischemia injury, respectively. These doses were therefore selected for the investigation into the effects of NaHS and PPG on acute myocardial ischemia injury. Three hours after the rats with acute myocardial ischemia were administered NaHS, the myocardial ultrastructural damage was significantly reduced, and increases in the NaHS dose led to more significantly reduced myocardial ultrastructural damage. This suggested that H_2_S could reduce acute myocardial ischemia injury and had a protective effect on myocardial structure subsequent to ischemia.

NF-κB, an important nuclear transcription factor, is widely found in eukaryotic cells and is a member of the Rel protein family. To date, five members of this family have been identified in mammals: p65 (RelA), RelB, C-Rel, p50/p105 (NF-κB1) and p52/p100 (NF-κB2) (32). These proteins are usually present in the form of homo-or heterodimers, wherein the heterologous dimer generated from p65 and p50 is the most common form. In a resting state, NF-κB binds with its inhibiting factor, inhibitor of NF-κB (IκB), and exists in a non-activated state in the cytoplasm. When the cells are under the influence of certain stimuli, such as ischemia, hypoxia, oxygen radicals, cytokines and certain viruses, IκB is phosphorylated, ubiquitinated, identified by the proteasome and then rapidly degraded, so as to expose the nuclear localization signal located on the p50 subunit. NF-κB is thus activated and translocates to the nucleus, where the transcription of numerous genes, including TNF-α, ICAM-1, cyclooxygenase-2, inducible nitric oxide synthase and phospholipase A_2_, is activated, (33,34). When myocardial ischemia occurs, vascular endothelial cells are stimulated first; following the activation of NF-κB the expression of a variety of substances, including TNF-α and vascular cell adhesion molecule-1 (VCAM-1), is initiated. Under the action of these neurotransmitters, leukocyte adhesion, migration, invasion and damage to the heart muscle appear in the ischemic region of the blood vessels. Further accumulation of white blood cells enhances the release of inflammatory mediators and oxygen radicals, which aggravate the ischemia, resulting in vascular and myocardial damage (35,36). In addition, the TNF-α neurotransmitter produced in the above process induces significant metabolic and hemodynamic changes in the body, and leads to an inflammatory factor ‘cascade effect’ (10). TNF-α can induce the generation of other inflammatory mediators, such as IL-1 and ICAM-1, and further activate NF-κB, so as to increase the degree of ischemic injury.

Cell adhesion molecules (CAMs) are a class of glycoprotein receptors present on the cell surface, and their main function is to promote cell-cell adhesion and cell-tissue matrix adhesion. CAMs play an important role in maintaining the stabilization of normal tissues, mediation of inflammatory responses, thrombosis, damage repair and immunoregulation (37). ICAM-1, a transmembrane protein antigen on the cell surface, is widely distributed in various tissues, and can activate T cells, endothelial cells, fibroblasts and tissue macrophages. There is only a low level of ICAM-l expression in myocardial cells under normal conditions, and its expression and activation are strictly regulated. Under the action of hypoxia and cytokines, large amounts of ICAM-1 are generated on the membrane surface of myocardial cells (38). NF-κB binding sites can be found on the ICAM-1 gene promoter (39): NF-κB is activated to enter the nucleus and promote the expression of ICAM-1; ICAM-1 can then in turn further activate NF-κB, thereby forming a positive feedback loop and continuously amplifying the inflammation.

IL-1, an inflammatory cytokine, is produced by activated leukocytes, particularly monocytes/macrophages, and is the initiating factor in the body’s inflammatory cytokine cascade; IL-1β is the main form of secretion. IL-1 may have a toxic effect through direct action on the cells, acting to destroy the structure and function of vascular endothelial cells and release large amounts of inflammatory cytokines, mediated by inflammatory cell adhesion, resulting in the excessive release of oxygen radicals, damaged vascular endothelial cells and decreased myocardial contractility. IL-1 can also activate platelets to stimulate platelet aggregation and thrombosis; in addition, it can produce vasoconstrictors, such as endothelin, to increase coronary vascular resistance (40). IL-6, an important inflammatory immune reaction medium, is involved in atherosclerosis formation and development, which is an important risk factor for coronary heart disease. IL-6 activation stimulates neutrophil and myocardial cell adhesion, so as to release plasmin and produce large amounts of oxygen radicals to damage myocardial cells. Simultaneously, IL-6 stimulates the expression of ICAM-1 on the surface of endothelial cells, leading to the increased permeability of the endothelium (41,42).

The results of the present study showed that, following acute myocardial ischemia, TNF-α, IL-1β and IL-6 levels in the serum in the myocardial tissue of rats were increased, and ICAM-1 mRNA and NF-κB expression in the myocardial tissues was significantly increased. Li *et al* (43) also reported that, following simple transient cardiac ischemia, NF-κB activity was rapidly and significantly increased; this may be the molecular mechanism underlying the rapid expression of a series of early inflammatory genes. The activation of NF-κB following myocardial ischemia could induce the production of TNF-α by myocardial tissues (36) in addition to regulating the expression of numerous genes, including IL-1β, IL-6, ICAM-l and VCAM-1. This indicates that the complex interaction of cytokines with NF-κB and inflammatory adhesion molecules can lead to further amplified and enhanced inflammation, resulting in myocardial inflammation, injury or even death.

This study showed that, following the administration of PPG, TNF-α, IL-1β and IL-6 levels in the serum and myocardial tissues of rats, as well as ICAM-1 mRNA expression and the expression of NF-κB protein, were increased. Following the administration of NaHS, however, TNF-α, IL-1β and IL-6 levels in the serum and myocardial tissues of rats, as well as ICAM-1 mRNA expression in myocardial tissues and NF-κB protein expression, were decreased, indicating that exogenously supplemented H_2_S inhibited the synthesis of inflammatory cytokines (such as IL-1β), nuclear transcription factors (such as TNF-α) and adhesion molecules in the serum and myocardial tissues of rats following the development of myocardial ischemia, thereby reducing myocardial injury and protecting myocardial tissues. In conclusion, the findings of the present study provide novel evidence for the exogenous supplement of H_2_S on acute myocardial ischemia injury via the modulation of inflammatory factors.

## Figures and Tables

**Table I:** Effect of hydrogen sulfide on the serum levels of TNF-α, IL-6 and IL-1β (n=6).

Group	TNF-α (ng/l)	IL-6 (ng/l)	IL-1β (ng/l)
Sham	84.03±12.49	194.36±26.32	48.67±4.50
Ischemia	168.47±17.13^a^	332.47±24.88^a^	86.79±7.82^a^
I + L NaHS	145.00±18.65	281.15±28.34^b^	74.41±7.43^b^
I + M NaHS	130.56±14.37^c^	260.15±30.94^c^	69.22±8.18^c^
I + H NaHS	114.93±17.85^c^	219.25±41.50^c^	61.32±7.34^c^
I + PPG	198.06±15.85^b^	384.71±37.55^b^	99.45±9.48^b^

Data are presented as the mean ± standard deviation.

a P<0.01 vs. the sham group;

b P<0.05 and

c P<0.01 vs. the ischemia group.

I + L NaHS, ischemia + 0.78 mg/kg NaHS; I + M NaHS, ischemia + 1.56 mg/kg NaHS; I + H NaHS, ischemia + 3.12 mg/kg NaHS; NaHS, sodium hydrosulfide; PPG, propargylglycine; TNF-α, tumor necrosis factor-α; IL, interleukin.

**Table II:** Changes in the expression of ICAM-1 mRNA and NF-κB protein in myocardial tissue in rats (n=5).

Group	ICAM-1 mRNA (relative content)	NF-κB (relative density)
Sham	0.42±0.05	0.72±0.062
Ischemia	0.98±0.10^a^	1.08±0.040^a^
I + L NaHS	0.83±0.08^b^	1.01±0.052
I + M NaHS	0.75±0.08^c^	0.98±0.033^c^
I + H NaHS	0.66±0.06^c^	0.90±0.036^c^
I + PPG	1.10±0.10	1.16±0.025^b^

Data are presented as the mean ± standard deviation.

a P<0.01 vs. the sham group;

b P<0.05 and

c P<0.01 vs. the ischemia group.

I + L NaHS, ischemia + 0.78 mg/kg NaHS; I + M NaHS, ischemia + 1.56 mg/kg NaHS; I + H NaHS, ischemia + 3.12 mg/kg NaHS; NaHS, sodium hydrosulfide; PPG, propargylglycine; ICAM-1, intercellular adhesion molecule-1; NF-κB, nuclear factor κ-light-chain-enhancer of activated B cells.
